# Adequate exercise response at artificial altitude in Fontan patients

**DOI:** 10.3389/fped.2022.947433

**Published:** 2022-08-18

**Authors:** Nicole Müller, Ulrike Herberg, Thomas Jung, Johannes Breuer, Julian Alexander Härtel

**Affiliations:** Department for Pediatric Cardiology, Children’s Hospital, University of Bonn, Bonn, Germany

**Keywords:** Fontan patients, artificial altitude, exercise capacity, physical activity, hemodynamic adaptation

## Abstract

**Purpose:**

For Fontan-palliated patients, altitude exposure is still a part of discussion since the extent of hypoxic pulmonary vasoconstriction potentially resulting in decreasing cardiac output (Qc), especially during physical exercise, is still unclear. We investigated the effects of normobaric hypoxia (15.2% O_2_) simulating 2,500 m above sea level on cardiopulmonary and metabolic parameters and the benefit of daily physical activity (PA) on hypoxic exercise capacity.

**Methods:**

A total of 21 Fontan patients (14–31 years) and 20 healthy controls performed cardiopulmonary exercise tests on a bicycle ergometer in normoxia and hypoxia until subjective exhaustion, measuring capillary lactate (cLa) every 2 min. In between, participants underwent an activity tracking over 5 days with a triaxial accelerometer.

**Results:**

Hypoxic exercise was well tolerated by Fontan patients, and no adverse clinical events were observed. Fontan patients showed reduced physical capacity under both conditions compared to controls (63% normoxia, 62% hypoxia), but the relative impairment due to hypoxia was similar for both (≈10%). Up to workloads of 2 W/kg oxygen uptake ($\dot{V}$O_2_) and heart rate (HR) developed similarly in patients and controls. cLa increased faster in relation to workload in Fontan patients, but remained significantly lower at peak workload (normoxia 3.88 ± 1.19 mmol/l vs. 7.05 ± 2.1 mmol/l; hypoxia 4.01 ± 1.12 mmol/l vs. 7.56 ± 1.82 mmol/l). Qc was diminished but could be increased similar to controls. Fontan patients with higher PA levels showed a higher $\dot{V}$O_2p*eak*_ in hypoxia.

**Conclusion:**

Exercise during short-time artificial altitude exposure seems to be safe for young Fontan patients. Further studies are needed to validate longer exposure under real conditions. $\dot{V}$O_2_, HR, and Qc might not be a limiting factor for exercise until workloads of 2 W/kg. Higher daily PA levels might improve physical capacity under altitude conditions.

## Introduction

High altitude is defined as an altitude of 2,500–3,500 m above sea level (masl). It results in reduced oxygen concentration and barometric pressure (1). As known from healthy individuals, hypoxia leads to vasoconstriction in the pulmonary arteries with the consequence of a rising pulmonary vascular resistance (PVR) in children and adults (2). For Fontan-palliated patients with a passive pulmonary perfusion due to a missing sub-pulmonary ventricle, a rising PVR was thought to lead to an impairment of pulmonary blood flow (PBF) and as a result low cardiac output (Qc) with limited mechanisms to compensate.

In Germany, every year ∼200 children are born with a complex heart defect, leading to a univentricular circulation (3). In the last four decades, a staged surgery has been established, resulting in Fontan palliation (4). This univentricular circulation currently enables a life expectancy until adulthood (5). But still for Fontan patients, life is different in many aspects. Rising up with a congenital heart defect (CHD) often means limitations in everyday life (6, 7).

Over the past decade, pediatric cardiologists and scientists have increasingly been able to establish that physical activity (PA) does not pose a danger to children with CHDs, but rather improves their physical performance (8–10). On this basis, pediatricians, as well as EMAH cardiologists, are confronted with the question of guidelines regarding all kinds of activities. Skiing, hiking, and mountain biking became popular sports for many families, but we do not yet know for sure whether in general altitude exposure alone or even PA at altitude is safe and feasible for patients with Fontan circulation.

Previous studies investigating the exercise capacity of patients after Fontan palliation under normal conditions at sea level (normoxia) showed a relevant impairment of exercise capacity with ∼60–65% of $\dot{V}$O_2p*eak*_ of age-matched healthy subjects (11–14). Besides cardiac function, including low Qc or chronotropic incompetence, further reasons for this impairment are multifactorial and include reduction in lung function, skeletal muscle, and endothelial dysfunction (15–18).

Studies by Garcia et al., Staempfli et al., and Takken et al. previously investigated hypoxic exposure to Fontan patients without any adverse events (19–21). Garcia analyzed data from a small group of 11 adolescent and young adult Fontan patients without matched controls, whereas Staempfli included only adult patients with single-ventricle physiology, both with a focus on PBF. Takken recently published data on teenagers and young adults, but all of them focused on peak performances. As endurance activities like hiking or skiing are not mainly absolved at peak physical capacity, this chapter focuses on differences at lower workloads as well as peak performances.

The aim of this study was to add further insights into physiological processes in Fontan patients at hypoxic exposure by investigating the impact of hypoxia on physical capacity and metabolic status, depending on daily PA. We therefore performed a study comparing performance parameters at different workloads in adolescent and young adult Fontan patients with matched healthy controls under normal conditions and in a normobaric hypoxic chamber, simulating an altitude of 2,500 masl (average height of European recreational mountain and skiing areas) and 5 days of activity tracking with a triaxial accelerometer.

## Materials and methods

### Participants

A total of 21 adolescent and young adult Fontan patients with a variety of underlying heart defects and 20 age, sex, and body mass index (BMI)-matched healthy controls were investigated in this study (for details, refer to Table 1).

**TABLE 1:** (a) Participants’ characteristics; (b) detailed description of patients and underlying congenital heart defects.

		Fontan (*n* = 21)	Control (*n* = 20)	Significance (*P*-value)
**(a)**				
Age	Years	18 [14,31]*	18 [14,33]*	ns
Gender	f/m	9/12	9/11	
Height	cm	166.3 ± 9.22	171.1 ± 9.9	ns
Body mass	kg	63.88 ± 14.23	66.25 ± 14.26	ns
Body mass index (BMI)	kg/m^2^	22.9 ± 3.77	22.26 ± 3.32	ns
Body surface area (Mosteller)	m^2^	1.71 ± 0.23	1.77 ± 0.23	ns
**(b)**				
Age at Fontan surgery	Month	41.9 ± 28.44		
Years since Fontan surgery	Years	15.76 ± 3.0		
Patients with implanted pacemakers		3		
**Underlying cardiac defect**				
*Tricuspid atresia*		3		
*Double inlet left ventricle (DILV)*		4		
*Single inlet left ventricle*		1		
*Hypoplastic left heart syndrome*		7		
*Double outlet right ventricle (DORV)*		2		
*Single ventricle with ccTGA*		4		
**Type of Fontan circulation**				
*Intraatrial tunnel*		15		
*Extracardiac tunnel*		6		
*Fenestration*		2		
**Medication**				
*Cardioselective β-blocker*		4		
*ACE-inhibitors*		8		
*Both*		2		

*Values are presented as median [min, max].

We defined the following inclusion criteria for Fontan patients: age ≥ 14 years at the time of investigation, New York Heart Association (NYHA) class I or II, as well as mental and physical ability for cardiopulmonary exercise testing (CPET) on a sitting bicycle ergometer.

Exclusion criteria were failing Fontan [with protein-losing enteropathy (PLE) and plastic bronchitis], $\dot{V}$O_2p*eak*_ < 45% of predicted values, and pregnancy.

The control group consisted of nonsmokers and noncompetitive athletes without cardiovascular disorders.

Approval was obtained from the local ethics committee of the University Hospital Bonn (application number 335/14), and the study conformed with the Declaration of Helsinki.

All participants/parents gave written informed consent before participating in the study.

The study was performed at the University Children’s Hospital Bonn, and data were collected from May 2018 to October 2018.

### Study procedure

#### Study design

Study participants had to absolve CPETs in normoxia and under hypoxic conditions since the influence of hypoxia should be demonstrated.

**FIGURE 1:**
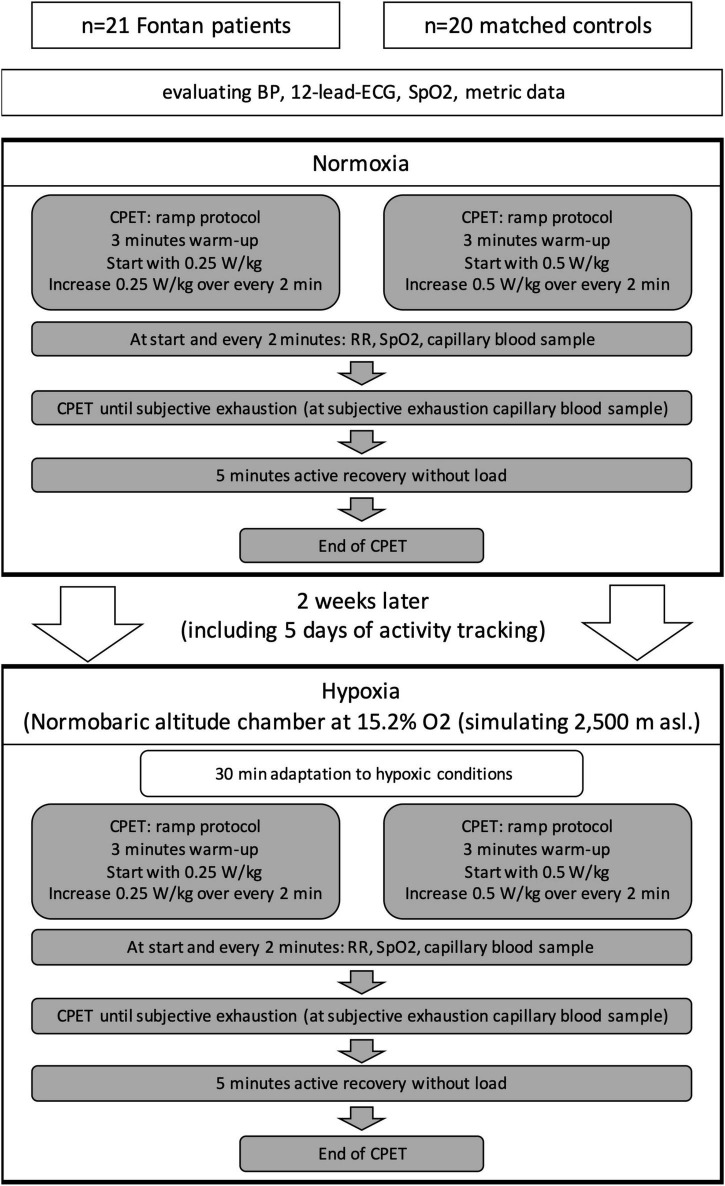
Study design.

Therefore, 2 days of examination were necessary and a period of 2 weeks was retained between the two examination days for safe recovery (for the detailed study protocol, refer also to Figure 1).

Examination days started with baseline evaluation of metric data, blood pressure, 12-lead ECG, and oxygen saturation. For the hypoxic conditions, all participants received a peripheral venous catheter for blood samples [e.g., measurement of hemoglobin (Hb)] and safety reasons. The patients had to fulfill the same protocol on both days in normoxia and under simulated altitude.

For baseline values of physical capacity, all investigations on the first visit were done under normoxic conditions, whereas investigations on the second day were performed in a fully climatized normobaric altitude chamber (Höhenbalance, Going, Austria) with stable temperature conditions between 21°C and 22.5°C. The size of the chamber was 12 m^2^ and contained ∼37.5 m^3^ air. Also, 60 m^3^ air was refreshed every hour. Ambient oxygen was reduced by membrane processes to a level of 15.2% (± 0.2%) and replaced by nitrogen (84.8%) at constant air pressure (1,013 hPa) simulating an altitude of 2,500 masl. When CO_2_ increased over 0.2%, fresh air was ducted into the altitude chamber. Participants started with 30 min of acclimatization, sitting on a chair after entering the hypoxic environment, to ensure realistic everyday conditions during hiking and skiing with rapid ascent by gondola, starting PA more or less right away. This was followed by the setting up for CPET.

In between the two examination visits, all participants received a triaxial accelerometer that they were encouraged to wear for at least 5 out of that 14-day period to get an overview of the participant’s everyday activity levels (see below).

#### Exercise testing

CPET was performed on a computer-controlled bicycle ergometer in an upright position (ERG 911 Plus, Ergosana GmbH Schiller, Bitz, Germany) with a breath-by-breath gas exchange analysis, which continuously measured tidal volumes (VT), respiration rate (RR), oxygen uptake ($\dot{V}$O_2_), and carbon dioxide production ($\dot{V}$CO_2_) (Metamax 3B with MetaSoft Studio Software v. 5.4 for analysis, Cortex Biophysik GmbH, Leipzig, Germany). The highest mean of oxygen uptake of any 30 s interval during exercise was defined as $\dot{V}$O_2p*eak*_.

Two different ramp protocols were chosen for either Fontan patients or controls to achieve similar loading times of ∼8–15 min. The end was defined by subjective exhaustion, according to the Borg scale ≥16 and lacking cadence ≥60 rpm. It was followed by a 5 min recovery period before finishing without load (Figure 1). For safety reasons under these special conditions, patients were not pushed further beyond their individual definition of exhaustion.

A continuous 12-lead ECG was monitored for heart rate (HR) and detection of potential arrhythmias (Custo Diagnostics, V. 5.4, Custo med GmbH, Ottobrunn, Germany). Blood pressure (BP_*sys*_, BP_*dia*_) was measured every 2 min (Tango M2, SunTech Medical Inc., Morrisville, United States). Transcutaneous oxygen saturation (SpO_2_) was continuously displayed by a pulse oximeter (V100, GE Healthcare, Chicago, United States). Capillary lactate probes (cLa) were taken from the earlobe and analyzed by enzymatic-amperometric measurement (LabTrend, BST Bio Sensor Technology GmbH, Berlin, Germany) within 2 h.

#### Impedance cardiography

Stroke volume (SV) and Qc were constantly recorded using the noninvasive thoracic impedance monitor PhysioFlow (Manatec Biomedical, Poissy, France), which was evaluated for patients with CHDs by Legendre et al. (22) and Ebrahim et al. (23). It was connected *via* Bluetooth to MetaSoft Studio Software. With still uncertain data regarding Fontan patients, we used this method to compare the two conditions (hypoxia and normoxia) in order to gain further clarity. The method was already used for this patient group in different studies (20, 24). As most patients have a history of veno-venous collaterals and knowing that this method can be influenced by many factors, especially shunt connections, we mainly focused on trends. Shunt volume was not quantified in more detail, and correlations with other parameters were not made.

#### Activity tracking

Between both examination visits, participants received a triaxial accelerometer, Actigraph wGT3x-BT (Actigraph LLC., Pensacola, United States), which *inter alia* was validated for reliable data by Lee et al. (25) and Chu et al. (26). The accelerometer was worn for at least 5 days, including one weekend day. The detailed analysis process was recently described (27).

Briefly, activity data with a sampling rate of 30 Hz were included when a minimum wear time of 330 min per day at five different days was reached.

Analysis was performed using the ActiLife 6 software (V.6.13.3, Actigraph LLC, Pensacole, United States). Different activity intensity categories were built by analyzing counts per minute with an epoch length of 10 s using the cutoff points by Freedson et al. and moderate or higher intensities were summarized as moderate-to-vigorous PA (MVPA) (28).

### Statistics

Statistical analysis was performed with GraphPad Prism (v.9.2.0, GraphPad Software, San Diego, CA, United States).

First, variables were tested for normal distribution using D’Agostino and Pearson normality test.

For investigation of normal distributed patients’ characteristics, Student’s unpaired *t*-test was performed.

To detect differences at rest and peak exercise under different conditions (normoxia vs. hypoxia) within and between groups, values were tested with one-way repeated-measures analysis of variance (ANOVA) and for *post hoc* analysis Tukey’s multiple comparison test was used.

Parameters at specific workloads between Fontan patients and controls and development of parameters within groups were compared using two-way ANOVA, followed again by Tukey’s multiple comparison test.

Correlation analysis between parameters was done with simple linear regression. To measure the correlation strength, Pearson’s correlation coefficient was applied.

Quantitative data are presented in mean ± standard deviation, unless otherwise described.

Level of significance was defined for *p*-values ≤ 0.05 (two-sided).

## Results

### Clinical outcome

All Fontan patients and matched controls could manage hypoxic conditions and exercise testing without any adverse events. During recovery in normoxia, one Fontan patient with known Kent bundle had an atrioventricular reentry tachycardia, requiring adenosine therapy.

One patient could not participate in the normoxia examinations due to time constraints, but was included in the calculations of the hypoxia results. The rest of the participants finished CPET under both conditions.

All relevant parameters at rest and peak exercise are listed in Table 2.

**TABLE 2:** Results of CPET in normoxia and hypoxia.

		Fontan patients			Controls					

Parameters	Units	Normoxia	Hypoxia	△ Hypoxia/normoxia	Normoxia	Hypoxia	△ Hypoxia/normoxia	*P*-value 1	*P*-value 2	*P*-value 3
**Rest**										
SpO_2_	[%]	92 ± 4	87 ± 5	−5.4%	99 ± 1	94 ± 2	−5.1%	<0.001*	<0.001*	<0.001*
HR	[bpm]	77 ± 12	69 ± 13	−10.4%	79 ± 10	70 ± 10	−11.4%	0.095	0.944	0.979
BP sys	mmHg	114 ± 10	114 ± 14		124 ± 13	123 ± 13		0.997	0.047	0.165
BP dia	mmHg	75 ± 11	72 ± 13		76 ± 11	73 ± 9		0.887	0.976	0.991
VT	[l]	0.61 ± 0.15	0.73 ± 0.16	19.7%	0.59 ± 0.23	0.65 ± 0.2	10.1%	0.327	>0.999	0.584
RR	[1/min]	21 ± 3	19 ± 3	−9.5%	18 ± 3	18 ± 5	0.0%	>0.999	0.129	0.11
V′O_2_	[l/min]	0.3 ± 0.07	0.34 ± 0.08	13.3%	0.33 ± 0.12	0.33 ± 0.1	2.4%	0.711	0.885	>0.999
V′O_2_/kg	[ml/min/kg]	4.93 ± 0.91	5.39 ± 1.27	9.3%	4.84 ± 1.4	5.11 ± 1.56	5.6%	0.668	0.997	0.897
V′O_2_/HR	[ml]	4.01 ± 1.02	4.34 ± 1.61	8.2%	4.17 ± 1.59	4.28 ± 1.51	2.6%	>0.999	>0.999	>0.999
V′E/V′CO_2_		40.51 ± 4.02	42.31 ± 5.5	4.4%	32.4 ± 4.81	32.99 ± 3.32	1.8%	0.579	<0.001*	<0.001*
SV	[ml]	52.96 ± 14.69	51.84 ± 14.57	−2.1%	68.10 ± 21.72	62.88 ± 13.33	−7.7%	>0.999	0.08	0.134
cLa	[mmol/l]	1.3 ± 0.4	1.35 ± 0.29	3.8%	1.29 ± 0.27	1.3 ± 0.19	0.8%	>0.999	>0.999	>0.999
pH		7.41 ± 0.03			7.39 ± 0.03				0.071	
BE		−1.52 ± 1.27			−0.07 ± 1.57				0.002*	
Hb	[g/dl]	15.55 ± 1.51			14.11 ± 0.98				<0.001*	
**VO_2_ peak**										
SpO_2_	[%]	89 ± 5	83 ± 7	−6.8%	97 ± 2	90 ± 3	−7.2%	<0.001*	<0.001*	<0.001*
HR	[bpm]	147 ± 29	145 ± 27	−1.4%	187 ± 13	185 ± 10	−1.1%	>0.999	<0.001*	<0.001*
VT	[l]	1.66 ± 0.46	1.64 ± 0.47	1.2%	2.24 ± 0.72	2.3 ± 0.73	2.7%	0.999	0.016*	0.004*
RR	[1/min]	37 ± 6	39 ± 6	5.4%	40 ± 8	42.1 ± 8	5.3%	0.719	0.515	0.586
P	[W]	112.7 ± 31.86	104.2 ± 29.8	−7.5%	209 ± 52.32	194.9 ± 45.72	−6.74%	0.91	<0.001*	<0.001*
P/kg	[W/kg]	1.81 ± 0.32	1.63 ± 0.24	−9.9%	3.19 ± 0.71	2.97 ± 0.61	−6.9%	0.681	<0.001*	<0.001*
V′O_2_	[l/min]	1.6 ± 0.44	1.44 ± 0.41	−10.0%	2.62 ± 0.7	2.42 ± 0.6	−7.6%	0.636	<0.001*	<0.001*
V′O_2_/kg	[ml/min/kg]	25.1 ± 4.49	22.71 ± 3.66	−9.5%	39.75 ± 9.6	36.6 ± 8	−7.9%	0.682	<0.001*	<0.001*
V′O_2_/HR	[ml]	10.8 ± 3.19	10.14 ± 2.69	−6.1%	14.1 ± 3.77	13.2 ± 3.65	−6.4%	>0.999	0.026*	0.033*
V′E/V′CO_2_		38.23 ± 4.35	43.01 ± 4.76	12.5%	30.73 ± 4.19	35.55 ± 4.07	15.7%	0.479	<0.001*	0.006*
Qc	[l/min]	10.33 ± 3.33	11.1 ± 3.08	7.8%	18.05 ± 5.2	16.93 ± 3.7	−6.2%	>0.999	<0.001*	<0.001*
SV	[ml]	71.5 ± 19.05	77.76 ± 18.64	8.8%	97.35 ± 28.67	92.2 ± 21.75	−5.3%	>0.999	0.01*	0.252
(a−v)DO_2_	[ml/100 ml]	8.61 ± 3.43	9.56 ± 2.27	11.0%	9.01 ± 2.44	8.99 ± 1.83	−0.2%	>0.999	>0.999	>0.999
cLa	[mmol/l]	3.88 ± 1.19	4.01 ± 1.12	3.4%	7.05 ± 2.1	7.56 ± 1.82	7.2%	0.993	<0.001*	<0.001*
BORG		19 ± 2	17 ± 2		20 ± 2	17 ± 2		>0.999	>0.999	>0.999
pH			7.34 ± 0.04			7.26 ± 0.05				<0.001*
BE			−5.87 ± 2.16			−8.49 ± 2.69				0.002*

p-value 1: differences within Fontan patients normoxia–hypoxia; p-value 2: differences between Fontan patients and controls normoxia; p-value 3: differences between Fontan patients and controls hypoxia. Significant differences are marked with*.

### Resting state

Significant differences at rest between normoxia and hypoxia in Fontan patients were only seen for SpO_2_ (Table 2).

In simulated altitude, the mean of SpO_2_ was 5 points lower than in normoxia. This decrease was also observed in controls even though the absolute SpO_2_ level was significantly higher under both conditions in this group. Furthermore, Fontan patients had significantly higher Hb.

HR in hypoxia was reduced in both groups compared to values in normoxia even though this effect was not statistically significant. A difference between Fontan patients and controls could not be seen neither in normoxia nor hypoxia.

Tidal volume increased under hypoxic conditions in both groups, while respiratory rate was similar and differences between Fontan patients and controls were not detected. Nevertheless, Fontan patients showed reduced respiration efficiency since $\dot{V}$E/$\dot{V}$CO_2_ was significantly increased compared to controls.

$\dot{V}$O_2_ and $\dot{V}$O_2_/kg were increased in hypoxia, even though the effect was not statistically significant. Values for Fontan patients and controls were similar.

SV measured by impedance cardiography was significantly reduced in Fontan patients under both conditions compared to controls, with no difference within the Fontan group in normoxia and hypoxia.

In none of the groups, baseline capillary lactate was increased after exposure to hypoxia.

### Peak exercise

Fontan patients achieved a peak workload (P_*peak*_) of 1.8 Watts/kilo bodyweight (W/kg) in normoxia compared to 1.6 W/kg in hypoxia. Under hypoxic conditions, $\dot{V}$O_2p*eak*_ decreased by ∼10% compared to normoxic values, corresponding to workloads. In contrast, controls showed a similar decrease (*p* = 0.453).

SpO_2p*eak*_ was significantly lower in Fontan patients in normoxia and hypoxia with a comparable decline on a higher level in controls.

Comparable to the resting state, $\dot{V}$E/$\dot{V}$CO_2_ was higher in the Fontan group, again showing a less efficient gas exchange also under peak exercise. The maximal oxygen pulse ($\dot{V}$O_2_/HR) did not vary within groups but differed significantly between Fontan patients and controls.

Arteriovenous oxygen difference (a-v)DO_2_ estimated by impedance cardiography did not show differences neither at maximum exhaustion in the Fontan group nor between groups, even though Fontan patients showed a trend for increasing (a-v)DO_2_ values in hypoxia.

SV_*peak*_ and Qc_*peak*_ showed reduced values compared to controls. Hypoxia did not lead to impaired Qc_*peak*_ in both groups. Furthermore, hypoxic exercise led to reduced SV_*peak*_ and Qc_*peak*_ in controls. For this, a statistically significant difference to the Fontan group could not be found [*p*_(SV)_ = 0.6064, *p*_(Qc)_> 0.9999].

Lactate levels were significantly lower in Fontan patients at $\dot{V}$O_2p*eak*_ with an incipient hyperacidity in the blood gas analysis mainly in the control group but also in Fontan patients, corresponding with a higher decline in base excess (BE).

### Submaximal exercise

**FIGURE 2:**
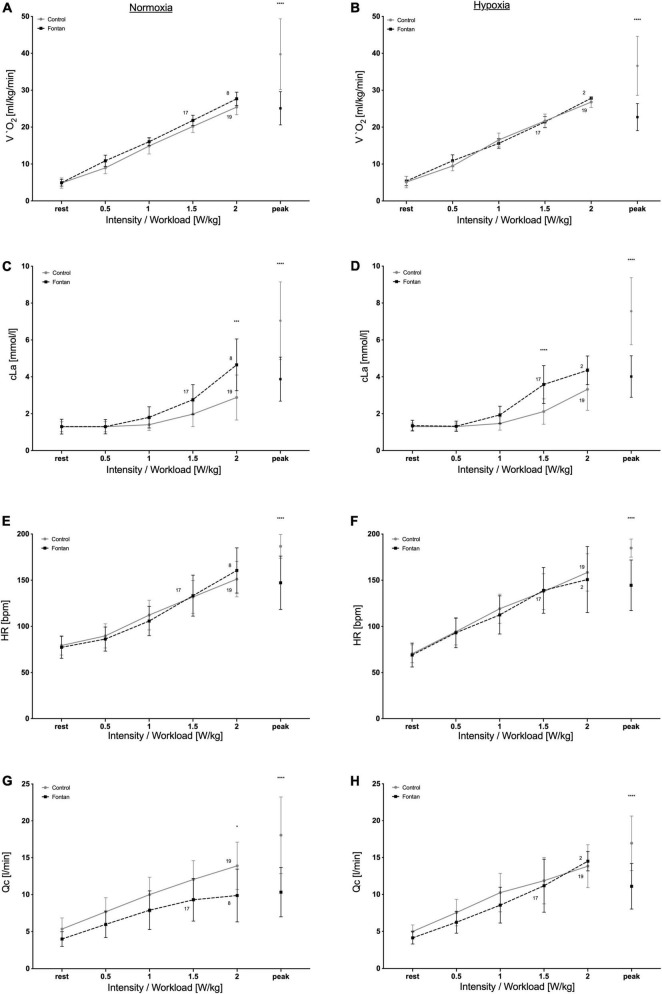
Development of cardiopulmonary and metabolic parameters during exercise in normoxia (left side) and hypoxia (right side). Fontan patients showed similar development of parameters except cLa, which already increased at lower workloads. Numbers show numbers of participants reaching the different workloads. **(A,B)** Development of $\dot{\text{V}}$O_2_, **(C,D)** development of cLa, **(E,F)** development of HR, and **(G,H)** development of Qc. Asterisks represent significance level: *p < 0.05; ***p < 0.001; ****p < 0.0001.

To achieve a better comparability of selected data, development of parameters during increasing workloads in W/kg, which is common for pediatric patients, is presented in Figure 2.

Only a minority of the Fontan patients reached workloads of 2 W/kg (8/21 in normoxia respective 2/21 in hypoxia).

$\dot{V}$O_2_ showed a similar increase until 2 W/kg (Figures 2A,B), with even slightly higher values in Fontan patients at similar workloads in normoxia, whereas in hypoxia values showed no differences. Correspondingly, HR increased parallel to $\dot{V}$O_2_ with no difference between groups, except for HR_*peak*_.

In contrast, cLa showed a steeper increase already at workloads of 1 W/kg compared to controls, even though $\dot{V}$O_2_ was similar (Figures 2C,D) under both conditions. While in normoxia, this difference was significant for values at workloads of 2 W/kg, in hypoxia a statistically significant difference was already seen for 1.5 W/kg.

Additionally, Qc showed a trend for reduced values in the patient group, even though the difference was not significant (Figures 2G,H). Interestingly, these developments were mainly seen in normoxia, whereas under hypoxic conditions Qc especially for workloads of 1.5 and 2 W/kg was similar.

### Moderate-to-vigorous physical activity and hypoxic physical capacity

A total of 18 Fontan patients and 16 controls fulfilled the criteria for analysis with sufficient wear time.

Differences in MVPA levels between Fontan patients and controls were not found, but both groups showed alarmingly low activity levels overall. With only 27% of Fontan teenagers and 33% of control teenagers, only a small proportion of this group reached the current WHO activity recommendations of 60 min MVPA/day, as recently shown (27).

**FIGURE 3:**
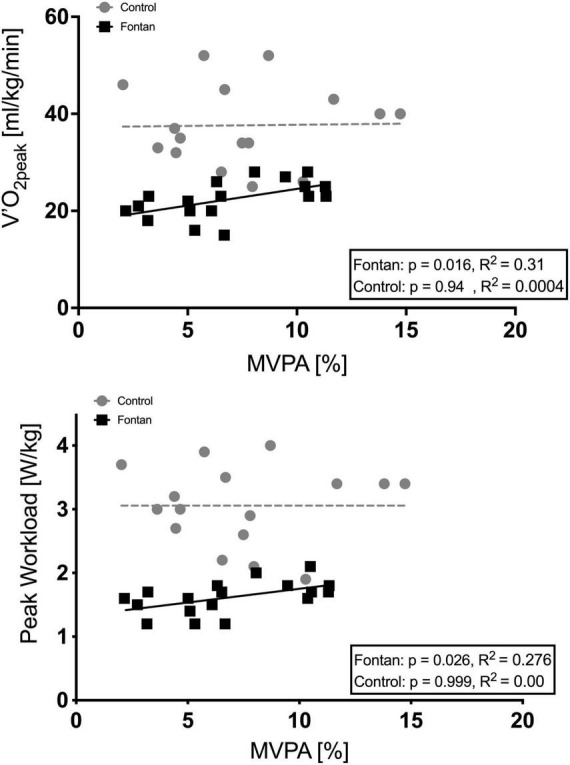
Correlation of MVPA (as percentage of time spent in MVPA) during everyday life with $\dot{\text{V}}$O_2p*eak*_ and MVPA with P_*peak*_ in hypoxia. A significant correlation was detected in Fontan patients.

In addition, a positive significant correlation could be demonstrated for MVPA with $\dot{V}$O_2p*eak*_ as well as for MVPA with P_*peak*_ in hypoxia in Fontan patients (Figure 3).

## Discussion

To the best of our knowledge, this is the first study showing the development of cardiopulmonary and metabolic parameters over different workloads during hypoxic exercise in Fontan patients and matched controls and thus complements important information to previous studies.

The Fontan circulation depends on a passive pulmonary perfusion since a sub-pulmonary ventricle is lacking. Therefore, factors increasing PVR such as hypoxic vasoconstriction are expected to be dangerous for these patients or at least have a further negative impact on physical capacity.

Compared to controls$,\dot{V}$O_2p*eak*_ and P_*peak*_ in normoxia were impaired in Fontan patients, which is in line with previous findings (8, 13). With 63% of the control value for $\dot{V}$O_2p*eak*_/kg in normoxia, respectively, 62% in hypoxia physical capacity stayed equal, so that the loss of physical capacity induced by hypoxia was similar between the groups. In Fontan patients and controls, $\dot{V}$O_2p*eak*_ decreased (10% in Fontan patients, 7.6% in controls) with no significant difference in effect size. In previous studies, a loss of 1% of $\dot{V}$O_2p*eak*_ was shown for every additional 100 m at altitudes higher than 1,500 masl for healthy individuals, resulting in an expected loss of $\dot{V}$O_2p*eak*_ of 10% at an altitude of 2,500 masl (29, 30). Consequently, hypoxia had a similar impact on this parameter in Fontan patients with no higher limitations, even though peak performance in Fontan patients was impaired. This result is compatible with the results of Staempfli et al. and Takken et al. but in contrast to these studies we could not demonstrate a significantly higher impact of hypoxia on controls (19, 20).

Furthermore, none of the patients showed any clinical symptoms during hypoxia exposure, even though SpO_2_ was significantly reduced at all-time points. The decline at SpO_2p*eak*_ was comparable between both groups under both conditions, which was also shown by Staempfli et al. (19) and Takken et al. (20).

Short maximal hypoxic exercise at altitudes of 2,500 masl therefore seems to be well tolerated by these patients. This finding is underlined by the fact that hypoxic pulmonary vasoconstriction (PVC) resulting in increasing PVR, which was expected to lead to reduced preload and disproportionally impairment in physical capacity, could not be seen.

This finding is also underlined by an increase in SV and Qc in both groups in normoxia and hypoxia (Table 2). As described earlier, this data obtained by impedance cardiography must be considered critically as shunt connections in particular can have a considerable influence on the method. As this study was intended to provide a general statement for Fontan patients, shunt connections were deliberately not an exclusion criterion. Thus, the absolute values of SV and Qc may be subject to greater deviations, while the trend development certainly allows for reliable information.

Furthermore, cLa did not increase at rest after 30 min of acclimatization and setting up for CPET in hypoxia and showed significant lower peak values in Fontan patients under both conditions, which might be explained by sufficient hypoxia tolerance as well as further cardiopulmonary capacities. We assume that $\dot{V}$O_2m*ax*_ was not reached by most participants, primarily because of muscular exhaustion of the legs, which was the main reason for termination of CPET. This might also explain the demonstrated low absolute cLa_*peak*_, especially in Fontan patients, since expectable cLa_*peak*_ at $\dot{V}$O_2m*ax*_ is reported to reach values of > 8–10 mmol/L (31). Corresponding to cLa_*peak*_, pH and BE were significantly reduced in controls compared to Fontan patients, which could also be explained by higher peak exercise in controls. Factors such as uncertainty in the unfamiliar environment and a lack of body awareness may play an additional role here. Many patients are not used to regular sport activities and show a low PA level so that they might not be used to the feeling of physical exhaustion (32).

In addition, physical exercise usually leads to an increase in (a-v)DO_2_ in healthy individuals since more oxygen is used for energy supply in the muscles. In our study, absolute (a-v)DO_2_ was lower compared to previously reported ranges from Ogawa et al. for healthy individuals under both conditions (33) with similar values for Fontan patients and controls. Again, the low (a-v)DO_2_ could be explained by a lack of objective cardiopulmonary exhaustion, but since values show similar results in normoxia and hypoxia in Fontan patients, an adequate oxygen supply at the point of subjective exhaustion under hypoxic conditions can be postulated. In the study of Takken et al. Fontan patients also showed reduced (a-v)DO_2_, but overall values were greater in his cohort, which could be explained by higher peak power. Furthermore, Takken described a decline under hypoxia, which is also in contrast to our findings (20), but in both cases (no difference vs. a decline compared to normoxia) the consequence is similar: oxygen supply is sufficient even under hypoxic conditions and low SpO_2_ with no rise in (a-v)DO_2_. Consequently, it can be speculated that Fontan patients are adapted to lower SpO_2_, which might be explained by significantly higher Hb, which is common and could also be proven in our collective (Table 2). Here, too, the influence of the potentially present shunt connections on the method must be taken into account since the (a-v) DO2 is also determined by impedance cardiography.

Previous altitude studies with Fontan patients only describe and compare parameters at peak exercise, but comparability to healthy controls is therefore only possible to a limited extent since Fontan patients only reach significantly lower P_*peak*_ (19–21). As skiing and hiking are popular sports for adolescents and young adults, it becomes an issue for Fontan patients as well as they want to be a recognized member of their peer groups and catch up with their classmates. Because these leisure activities are predominantly in the aerobic range, the focus of the evaluation was placed on the areas of light and moderate PA. Hence, this study demonstrates important insights into the development of cardiopulmonary and metabolic parameters below $\dot{V}$O_2p*eak*_ (Figure 2).

Exposition to acute hypoxia leads to reduced oxygen supply in the lungs, resulting in hypoxemia with the consequence of increasing ventilation, HR, and Qc within minutes (34). Interestingly, in this study we found a trend of reduced HR and respiratory rate in hypoxia, which could be explained by our study protocol, since all participants started examinations in normoxia and therefore might have been more agitated, as described above.

After starting CPET, important exercise parameters, such as $\dot{V}$O_2_, HR, and Qc, showed similar increases and the repeatedly discussed chronotropic incompetence could not be demonstrated up to workloads of 2.0 W/kg. This is in line with the findings by Claessen et al. and Hedlund et al., who also demonstrated regular HR response and speculated that reduced HR_*peak*_ in Fontan patients might be an expression of a physiological reaction to maintain SV and Qc within the context of Frank–Starling mechanism, because of reduced preload (35, 36). In addition, due to the numerous operations, many Fontan patients have a lack of responsiveness to modulation by the autonomic nervous system and an impaired sinus node function, both with the consequence of a limited HR variability and HR reserve (13, 37, 38). Here, similar to previous studies, Fontan patients showed significantly lower HR_*peak*_ compared to controls, but these values were measured at significant lower P_*peak*_ (39). Therefore, a statement to reduced preload resulting in impaired HR_*peak*_ is not possible because exercise response to increasing workloads was adequate for study participants.

As performance limitations of Fontan patients are still a part of discussion (40, 41), this study demonstrated the good ability of cardiopulmonary adaptation to both exercise and hypoxia in these patients with no differences to controls. A reason for the significant earlier termination of CPETs could not be explained by cardiopulmonary parameters, except cLa.

Even though $\dot{V}$O_2_ was similar for workloads until 2.0 W/kg, cLa increased earlier according to workloads in the Fontan group than in healthy subjects, but stayed significantly lower at P_*peak*_ in normoxia as well as in hypoxia (Figure 2). A possible explanation might be the differences in muscle metabolism, muscle blood flow, and/or muscle fiber composition compared to controls, complemented by significantly reduced SpO_2_. Cordina et al. demonstrated reduced skeletal muscle mass and a poorer aerobic muscle capacity because of impaired phosphocreatine resynthesis (18). Furthermore, Turquetto et al. reported decreased muscle volume and blunted blood supply (42). All explanations could lead to reduced exercise tolerance with increased anaerobic energy consumption.

Anyhow, the earlier termination at significantly lower cLa_*peak*_ could be due to lower training status and poorer tolerance of muscle soreness during increasing lactate levels since previous studies showed lower levels of PA than recommended for the majority of Fontan patients and reduced participation in sports club activities, as mentioned above (32, 43). In addition, Fontan patients often avoid activities in vigorous intensity because of concerns about cardiopulmonary tolerance, arrhythmic events or restrictions imposed by parents and doctors, as shown by Rhodes et al. (44). In this study, we did not find significant differences between MVPA in Fontan patients and controls, but in both groups MVPA was alarmingly low and a positive correlation was demonstrated for MVPA with exercise parameters such as $\dot{V}$O_2p*eak*_ or with cLa (27). In addition, we could demonstrate that the frequency of MVPA during everyday life activities has a positive impact on hypoxic exercise tolerance in Fontan patients. This is certainly an important finding, which should be considered when counseling Fontan patients, especially when it comes to active stays in the mountains. These results become even more important because recently published studies claim reduced activity levels during the corona pandemic, especially in children with CHD (45). A promotion for regular PA and a regular training should therefore be implemented in a multimodal concept for long-term care of Fontan patients to maintain and improve exercise capacity, quality of life, and survival.

Consequently, since the development of important cardiopulmonary parameters like Qc, HR, and $\dot{V}$O_2_ during hypoxic exercise is similar in Fontan patients and healthy controls and even short but exhausting exercise at 15.2% O_2_ is well-tolerated, the induced hypoxia does not seem to cause a disproportionate increase in PVC, leading to a relevant reduction in PBF. The continuous, non-pulsatile blood flow in pulmonary arteries might result in endothelial dysfunction and/or vascular remodeling, with lack of PVC as already discussed by Turquetto et al. (42) and Kodama et al. (43). Nevertheless, further studies are needed to investigate PBF and pulmonary artery pressure (PAP) under hypoxic conditions since all the demonstrated results only measure PBF indirectly.

## Conclusion

In conclusion, normobaric hypoxia (15.2% O_2_) combined with short-term exercise is well-tolerated by “healthy” Fontan patients. Although peak exercise is diminished, altitude conditions have a similar impact on physical capacity compared to controls. Important exercise parameters such as $\dot{V}$O_2,_ HR, SV, or Qc indicate a comparable development in both groups until workloads of 2.0 W/kg and consequently reasons for exercise limitations could not be demonstrated for cardiopulmonary parameters. Nevertheless, cLa showed a significant earlier increase, which might be explained by impairments in skeletal musculature and/or poor training status. Physicians and parents should therefore promote PA since higher MVPA levels even during everyday life might be important to maintain and improve physical capacity also for exercise at altitude, quality of life, and long-term survival.

### Limitations

In this study as well as in previous studies by Staempfli et al., Takken et al., and Garcia et al. only the effects of acute hypoxic exercise were tested. Recommendations for trips with overnight stays at altitude therefore cannot be derived and need further investigation, especially since respiration while sleeping cannot be controlled and might result in dangerous desaturation.

The small sample size with restriction to Fontan patients with NYHA class I–II does not allow a general advice for all Fontan patients. Underlying CHD leading to a Fontan circulation varies vast and also health status is very different between patients. Therefore, individualized investigations for altitude tolerance should be recommended before altitude exposure.

The majority of Fontan patients quit CPET because of muscle exhaustion, and therefore might not have reached their individual maximal physical capacity. Nevertheless, values of P_*peak*_ and $\dot{V}$O_2p*eak*_ as well as HR_*peak*_ are in line with previous studies.

PBF and PVC were only measured indirectly by Qc or $\dot{V}$O_2_. Therefore, measurement of PAP would be essential for a better understanding of PVC and the adaptation to hypoxia.

Due to the study design, all participants had to start with the examination in normoxia, which might have led to differences in some of the investigated parameters due to excitement and uncertainty. Nevertheless, during exercise this effect becomes less important.

This study was performed in a normobaric altitude chamber where effects might be different. Millet et al. demonstrated different physical reactions for normobaric and hypobaric hypoxia (46). Still, normobaric hypoxia is used in many studies because it represents a good approximation to reality and enables a high level of safety for the patients. In real altitude, factors like humidity and temperature fluctuations have to be considered, but the impact of these issues was not the topic of investigation and might play an additional role during stays in the mountains.

## Data availability statement

The datasets presented in this article are not readily available because the ethical approval and the informed consent of the participants and the parents in this study do not cover any transfer of raw sensitive data to external sites. Therefore, the sensitive data supporting the findings of this study are only available on reasonable request from the institutional study nurse of the Department for Paediatric Cardiology of the University Hospital Bonn, who is in charge for data administration. Requests to access the datasets should be directed to Caroline von dem Bussche, Caroline.von_dem_Bussche@ukbonn.

## Ethics statement

The studies involving human participants were reviewed and approved by the Local Ethics Committee of the University Hospital Bonn (application number: 335/14). Written informed consent to participate in this study was provided by the patients/participants’ legal guardian.

## Author contributions

NM, JH, UH, and JB: conceptualization. NM, TJ, and JH: formal analysis. NM, UH, TJ, and JH: investigation. NM and JH: writing—original draft. UH and JB: writing—review and editing. All authors contributed to the article and approved the submitted version.
